# Supplementary material for: HIVprotI: an integrated web based platform for prediction and design of HIV proteins inhibitors
Source: J Cheminform. 2018 Mar 9;10:12. doi: 10.1186/s13321-018-0266-y (PMC5845081; doi:10.1186/s13321-018-0266-y)
Supplement: Supplementary file 2 — Additional file 2. Source code of HIVProtI web server. [file 13321_2018_266_MOESM2_ESM.zip › HIVprotI_Source-code/hivprotI/weka-3-6-6/doc/weka/core/xml/MethodHandler.html]

MethodHandler


---


|  |  |  |
| --- | --- | --- |
| **Overview** \| **Package** \| **Class** \| **Tree** \| **Deprecated** \| **Index** \| **Help** \| **Weka's home** | |  |
| **PREV CLASS**   **NEXT CLASS** | **FRAMES**    **NO FRAMES**     **All Classes** |  |
| SUMMARY: NESTED \| FIELD \| CONSTR \| METHOD | DETAIL: FIELD \| CONSTR \| METHOD |  |


---


## weka.core.xml Class MethodHandler

```
java.lang.Object
  weka.core.xml.MethodHandler
```

**All Implemented Interfaces:**: RevisionHandler

---

``` public class MethodHandler extends java.lang.Object implements RevisionHandler ```

This class handles relationships between display names of properties
(or classes) and Methods that are associated with them.

**Version:**
:   $Revision: 1.3 $

**Author:**
:   FracPete (fracpete at waikato dot ac dot nz)

---

**Constructor Summary**

`MethodHandler()`
  
          initializes the handler


| **Method Summary** | |
| --- | --- |
| `void` | `add(java.lang.Class c, java.lang.reflect.Method method)`             adds the specified method for the given class to its internal list. |
| `void` | `add(java.lang.String displayName, java.lang.reflect.Method method)`             adds the specified method for the property with the given displayname to its internal list. |
| `void` | `clear()`             removes all mappings |
| `boolean` | `contains(java.lang.Class c)`             checks whether a method is stored for the given class |
| `boolean` | `contains(java.lang.String displayName)`             checks whether a method is stored for the given property |
| `java.lang.reflect.Method` | `get(java.lang.Class c)`             returns the stored method for the given class |
| `java.lang.reflect.Method` | `get(java.lang.String displayName)`             returns the stored method for the given property |
| `java.lang.String` | `getRevision()`             Returns the revision string. |
| `java.util.Enumeration` | `keys()`             returns an enumeration over all currently stored custom methods, i.e. |
| `boolean` | `remove(java.lang.Class c)`             removes the method for the specified class from its internal list. |
| `boolean` | `remove(java.lang.String displayName)`             removes the method for the property specified by the display name from its internal list. |
| `int` | `size()`             returns the number of currently stored Methods |
| `java.lang.String` | `toString()`             returns the internal Hashtable (propety/class - method relationship) in a string representation |

**Methods inherited from class java.lang.Object**

`equals, getClass, hashCode, notify, notifyAll, wait, wait, wait`

**Constructor Detail**

### MethodHandler

```
public MethodHandler()
```

:   initializes the handler


**Method Detail**

### keys

```
public java.util.Enumeration keys()
```

:   returns an enumeration over all currently stored custom methods, i.e. it
    returns the display names/classes in the enumeration.

    :   **Returns:**: the currently stored methods **See Also:**: `m_Methods`

---


### add

```
public void add(java.lang.String displayName,
                java.lang.reflect.Method method)
```

:   adds the specified method for the property with the given displayname
    to its internal list.

    :   **Parameters:**: `displayName` - the display name of the property to handle manually: `method` - the method, which will be invoked by reflection to handle the property manually **See Also:**: `m_Methods`

---


### add

```
public void add(java.lang.Class c,
                java.lang.reflect.Method method)
```

:   adds the specified method for the given class to its internal list.

    :   **Parameters:**: `c` - the class to handle manually: `method` - the method, which will be invoked by reflection to handle the property manually **See Also:**: `m_Methods`

---


### remove

```
public boolean remove(java.lang.String displayName)
```

:   removes the method for the property specified by the display name
    from its internal list.

    :   **Parameters:**: `displayName` - the display name of the propery to remove the custom method for **Returns:**: whether the method was stored in the list at all **See Also:**: `m_Methods`

---


### remove

```
public boolean remove(java.lang.Class c)
```

:   removes the method for the specified class from its internal list.

    :   **Parameters:**: `c` - the class to remove the custom method for **Returns:**: whether the method was stored in the list at all **See Also:**: `m_Methods`

---


### contains

```
public boolean contains(java.lang.String displayName)
```

:   checks whether a method is stored for the given property

    :   **Parameters:**: `displayName` - the display name of the property to check for a method **Returns:**: whether a method is currently stored **See Also:**: `m_Methods`

---


### contains

```
public boolean contains(java.lang.Class c)
```

:   checks whether a method is stored for the given class

    :   **Parameters:**: `c` - the class to check for a method **Returns:**: whether a method is currently stored **See Also:**: `m_Methods`

---


### get

```
public java.lang.reflect.Method get(java.lang.String displayName)
```

:   returns the stored method for the given property

    :   **Parameters:**: `displayName` - the display name of the property to retrieve the method for **Returns:**: the method associated with the display name, can be `null` **See Also:**: `m_Methods`

---


### get

```
public java.lang.reflect.Method get(java.lang.Class c)
```

:   returns the stored method for the given class

    :   **Parameters:**: `c` - the class to retrieve the method for **Returns:**: the method associated with the class, can be `null` **See Also:**: `m_Methods`

---


### size

```
public int size()
```

:   returns the number of currently stored Methods

    :   **Returns:**: the nummber of methods

---


### clear

```
public void clear()
```

:   removes all mappings

---


### toString

```
public java.lang.String toString()
```

:   returns the internal Hashtable (propety/class - method relationship) in
    a string representation

    :   **Overrides:**: `toString` in class `java.lang.Object`
    :   **Returns:**: the object as string

---


### getRevision

```
public java.lang.String getRevision()
```

:   Returns the revision string.

    :   **Specified by:**: `getRevision` in interface `RevisionHandler`
    :   **Returns:**: the revision


---


|  |  |  |
| --- | --- | --- |
| **Overview** \| **Package** \| **Class** \| **Tree** \| **Deprecated** \| **Index** \| **Help** \| **Weka's home** | |  |
| **PREV CLASS**   **NEXT CLASS** | **FRAMES**    **NO FRAMES**     **All Classes** |  |
| SUMMARY: NESTED \| FIELD \| CONSTR \| METHOD | DETAIL: FIELD \| CONSTR \| METHOD |  |


---
